# Adoption of Rapid Diagnostic Tests for the Diagnosis of Malaria, a Preliminary Analysis of the Global Fund Program Data, 2005 to 2010

**DOI:** 10.1371/journal.pone.0043549

**Published:** 2012-08-27

**Authors:** Jinkou Zhao, Marcel Lama, Eline Korenromp, Patrick Aylward, Estifanos Shargie, Scott Filler, Ryuichi Komatsu, Rifat Atun

**Affiliations:** 1 The Global Fund to Fight AIDS, Tuberculosis and Malaria, Geneva, Switzerland; 2 Jiangsu Provincial Center for Disease Control and Prevention, Nanjing, China; 3 Erasmus University Medical Centre, Rotterdam, The Netherlands; 4 Imperial College, London, United Kingdom; Kenya Medical Research Institute - Wellcome Trust Research Programme, Kenya

## Abstract

**Introduction:**

The World Health Organization Guidelines for the Treatment of Malaria, in 2006 and 2010, recommend parasitological confirmation of malaria before commencing treatment. Although microscopy has been the mainstay of malaria diagnostics, the magnitude of diagnostic scale up required to follow the Guidelines suggests that rapid diagnostic tests (RDTs) will be a large component. This study analyzes the adoption of rapid diagnostic testing in malaria programs supported by the Global Fund to fight AIDS, Tuberculosis and Malaria (Global Fund), the leading international funder of malaria control globally.

**Methods and Findings:**

We analyzed, for the period 2005 to 2010, Global Fund programmatic data for 81 countries on the quantity of RDTs planned; actual quantities of RDTs and artemisinin-based combination treatments (ACTs) procured in 2009 and 2010; RDT-related activities including RDTs distributed, RDTs used, total diagnostic tests including RDTs and microscopy performed, health facilities equipped with RDTs; personnel trained to perform rapid diagnostic malaria test; and grant budgets allocated to malaria diagnosis. In 2010, diagnosis accounted for 5.2% of malaria grant budget. From 2005 to 2010, the procurement plans include148 million RDTs through 96 malaria grants in 81 countries. Around 115 million parasitological tests, including RDTs, had reportedly been performed from 2005 to 2010. Over this period, 123,132 health facilities were equipped with RDTs and 137,140 health personnel had been trained to perform RDT examinations. In 2009 and 2010, 41 million RDTs and 136 million ACTs were purchased. The ratio of procured RDTs to ACTs was 0.26 in 2009 and 0.34 in 2010.

**Conclusions/significance:**

Global Fund financing has enabled 81 malaria-endemic countries to adopt WHO guidelines by investing in RDTs for malaria diagnosis, thereby helping improve case management of acute febrile illness in children. However, roll-out of parasitological diagnosis lags behind the roll-out of ACT-based treatment, and will require prioritization of investments.

## Introduction

Malaria continues to exert a heavy disease and death burden, despite being preventable and treatable [1]. While parasite-based diagnosis of malaria is increasing, most suspected cases are treated following presumptive diagnosis, resulting in unnecessary over-use of antimalarial drugs for non-malaria febrile illness, and an inability to effectively track true malaria morbidity [2].

In 2006, WHO issued its first Guidelines for the Treatment of Malaria [3]. Since then, most countries where *Plasmodium (P.) falciparum* is endemic have progressively updated treatment policies to shift from failing chloroquine (CQ) and sulfadoxine-pyrimethamine (SP) to the WHO-recommended artemisinin-based combination therapies (ACTs). In its second edition [4] of the Guidelines in 2010, WHO additionally?recommended parasitological confirmation by microscopy or RDT for all patients suspected of malaria, including children under-5 years, before starting treatment; treatment solely on the basis of clinical suspicion is to be considered only when a parasitological diagnosis is not accessible [4].

The roll-out of RDTs will greatly enhance the quality of malaria diagnosis and treatment in many high-endemic areas, and will decrease over-use of ACTs in patients with non-malarial fevers, as demonstrated in studies undertaken in Senegal and Tanzania [5], [6]. Improved diagnostic coverage with RDTs should also improve malaria surveillance.

As with other health innovations, a range of contextual and health system factors [6]–[10] as well as clinical and health service readiness are likely to influence the adoption of the new case management guidelines. Although the proportion of suspected cases in Africa who received a malaria diagnostic test has risen from 20% in 2005 to 45% in 2010, RDT coverage remains low [1], and many malaria-affected countries especially in Africa continue the outdated, harmful practice of presumptively treating fever cases as malaria [1], [11]–[13].

Microscopy remains the reference method for malaria diagnosis, but many endemic and non-endemic countries lack expertise in microscopy [2], [14]. Malaria RDTs were introduced in the 1990s [14]. Since then, more than 60 RDT brands and over 200 different products have been developed [15], with improving sensitivity and specificity. Since the publication of WHO’s 2006 Guidelines, many countries have begun to expand RDT usage. However, the extent of this expansion is not known, as routine health information systems do not always distinguish between microscopy and RDT use.

The Global Fund to fight AIDS, Tuberculosis and Malaria (Global Fund) began investing in malaria control in 2002. By 2010 Global Fund investments accounted for around 75% of global external funding for malaria [16], complementing financing by the United States President’s Malaria Initiative (PMI), which, since 2005, has been a significant donor for malaria control programs in 19 countries in Africa and the Greater Mekong sub-region in Asia. These external investments have provided recipient countries the opportunity to accelerate the adoption of recommended malaria case management policies. We analyzed Global Fund investments across 81 countries with malaria grants for the period 2005 to 2010 to ascertain uptake of RDTs, and health service readiness for their deployment.

## Methods

### Program Data Source and Collection

We used data from Global Fund-supported malaria programs (Box S1) covering the period 2005 to 2010 for, (i) Country Procurement and Supply Management (PSM) plans by country and grant that identify the quantities of RDTs to be procured; (ii) Price and Quality Reporting (PQR) system, which includes data reported by Global Fund recipients on health products procured including RDTs and ACTs (http://www.theglobalfund.org/en/procurement/pqr/); (iii) Grant indicator results related to RDTs, extracted from Performance Frameworks of each grant, namely RDTs distributed, RDTs used, RDTs used *or* microscope tests performed (from grants that reported RDT and microscopy results as one overall combined number; Table 1), health facilities equipped with RDTs, and personnel trained to perform RDTs; (iv) Financial data on the amounts budgeted in grants for malaria diagnosis.

**Table 1:** Programmatic results reported by Global Fund malaria grant recipients on planning, distribution and use of rapid diagnostic tests (RDTs), parasitological confirmation and health service readiness over the years 2005 to 2010.

Indicator	Year					
	2005	2006	2007	2008	2009	2010
RDT procurements planned	3,825,974	8,172,790	20,424,441	28,291,346	38,579,902	48,971,130
RDTs procured	n/a	n/a	n/a	n/a	16,414,741	24,511,732
RDTs distributed	971,897	1,602,475	10,884,534	11,313,742	17,138,843	19,219,064
RDTs used	1,038,412	1,102,475	2,230,784	1,839,342	2,982,413	8,523,366
RDTs used or microscopy slides read	63,426	2,082,075	11,553,359	17,090,755	31,951,853	34,214,896
Total of RDTs used and microscopy slides read	1,101,838	3,184,550	13,784,143	18,930,097	34,934,266	42,738,262
Health facilities equipped with RDTs	1,996	20,174	23,049	13,606	31,947	32,360
Health personnel trained to perform RDTs	807	9,034	12,970	44,146	16,303	63,880

As grants use different indicators are used to report RDT-related activities to the Global Fund [17], the reported results are individually checked and aligned to the calendar years when the results are actually reported.

### Malaria Epidemiology Data

We obtained global and country-specific epidemiological data for malaria from WHO World Malaria Reports from 2006 to 2010. We used malaria endemicity categorization proposed in the World Malaria Report 2008 [18] to group countries as low or high endemicity, defined as below or equal and above 50 malaria cases per 1,000 population.

### Data Analysis

We used PSM plan data aggregated by year and geographic regions, and grouped by year. PQR-reported RDT and ACT procurements were tabulated and the ratio of volumes of RDTs to treatment courses calculated. Both the totals and the ratio are reported as results. We aggregated program implementation results by year, geographic region and malaria endemicity. Regional analysis stratified data according to Global Fund regions [19], into Africa (East Africa and Indian Ocean, Southern Africa, West and Central Africa, Middle East and North Africa), Asia (East Asia and the Pacific, South and West Asia); and the rest of world (Eastern Europe and Central Asia, Latin America and Caribbean). Analysis was done using SPSS 13.0 (SPSS Inc., Chicago, IL).

## Results

196 malaria grants in 81 countries had secured funding from the Global Fund through Rounds 1 to 9. By 2010, the Global Fund had committed US$5.3 billion to malaria programs in these countries. In 2010, 5.2% of the budgets of these grants were allocated to diagnosis, including RDTs [19].

Over 2005 to 2010, 96 malaria grants in the 81 countries had procurement plans that included an RDT component; 62 grants in 52 countries reported actual RDT procurement data into the PQR in 2009 or 2010; 87 grants from 76 countries reported program implementation results for malaria diagnosis.

### PSM Plans and PQR Data

**Figure 1:**
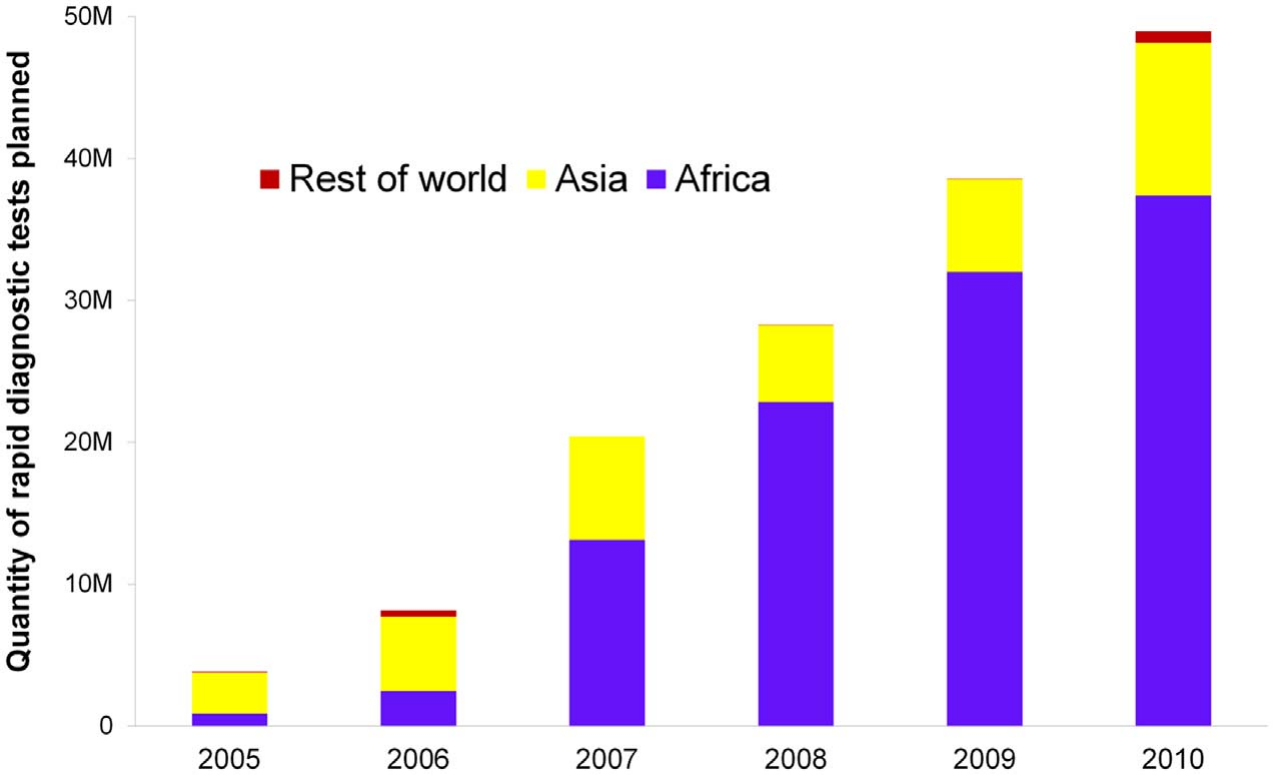
Planned procurement of rapid diagnostic tests funded by the Global Fund, 2005 to 2010.

Over 2005 to 2010, 96 grants planned to procure more than 148 million RDTs. Compared with 2005, numbers of RDTs included in PSM plans doubled in 2006 and increased substantially afterwards, reaching almost 50 million in 2010. Increases were observed in Africa and Asia from 2006 (Figure 1). Actual procurements were 16.4 million and 24.5 million, in 2009 and 2010 respectively (Table 1). In comparison, 63.9 million and 72.1 million treatment courses of ACTs were procured in 2009 and 2010. The ratio of RDTs procured relative to ACTs procured thus was low, although it increased from 0.26 in 2009 to 0.34 in 2010.

### Program Implementation Results

Cumulatively, 61.1 million RDTs were distributed from 2005 to 2010 through Global Fund-supported malaria programs (Table 1). The majority of RDTs were distributed in Africa (51.3 million, 83.9%) and in countries of high malaria endemicity (48.6 million i.e. 79.5%) (Tables 2 and 3).

**Table 2:** Global Fund programmatic results on distribution, use of RDTs and microscopy, and health service readiness, by region, 2005 to 2010.

Indicators	Region		
	Africa	Asia	Rest of the world
RDTs distributed	51,308,208	9,748,748	73,599
RDTs used	8,883,380	8,759,813	73,599
RDTs used or microscope slides read	46,607,733	39,112,276	11,236,355
Total of RDTs used and microscopy slides read	55,491,113	47,872,089	11,309,954
Health facilities equipped with RDTs	82,160	40,568	404
Health personnel trained to perform RDTs	119,622	11,640	15,878

**Table 3:** Global Fund programmatic results on distribution, use of RDTs and microscopy, and health service readiness, by malaria endemicity, 2005 to 2010.

Indicator	Endemicity: estimated cases per 1,000 population’ [18]			
	0–4	5–49	50–200	≥201
RDTs distributed	526,721	12,032,989	19,718,579	28,852,266
RDTs used	593,236	10,717,489	169,236	6,236,831
RDTs used or microscope slides read	36,787,917	14,889,139	21,852,749	23,426,559
Total of RDTs used and microscopy slides read	37,381,153	25,606,628	22,021,985	29,663,390
Health facilities equipped with RDTs	5,968	36,054	75,703	5,407
Health personnel trained to perform RDTs	21,380	3,869	99,058	22,833

RDT distributions increased from 971,900 in 2006 to 19.2 million in 2010 (Table 1). In 2009, RDT distributions equalled PQR-reported RDT procurements (16.4 million), suggesting rapid distribution of RDTs after procurement. In 2010, RDT distributions were close to 80% the volume of RDT procurements (19.2 million and 24.5 million respectively) (Table 1).

From 2005 to 2010, 114.7 million parasitological tests were reportedly done, including 17.7 million ‘RDTs tests’ alone and an additional 97.0 million ‘RDTs tests or microscope slides’ as a combined indicator. About half of these tests (55.5 million; 50.1%) were reported by countries in Africa, close to half (47.9 million; 49.4%) by Asian countries (Table 2), and 63.8% (62.9 million) by countries of low malaria endemicity (Table 3).

The number of RDTs used increased substantially, from 1.04 million in 2005 to 2.98 million in 2009 and 8.52 million in 2010 (Table 1). Nearly all (96.9 million, 98.0%) ‘microscope slides read or RDTs used’ were reported in 2007 or later (Table 1). Total parasitological tests administered increased by 42 times, from 1.1 million in 2005 to 42.7 million in 2010.

Between 2005 and 2010, 123,132 health facilities were reported to have been equipped with RDTs, and 147,140 health personnel were trained to perform RDTs (Tables 1, 2 and 3). Most health facilities equipped and personnel trained were in Africa (82,160 or 66.7% and 119,622 or 81.3%), in high-endemic countries (81,110 or 66.0%, and 121,891 or 83.0%).

## Discussion

The analysis shows a substantial increase in availability and use of RDTs in Global Fund supported malaria programs including RDTs procured and distributed, number of tests performed, and health service readiness to undertake diagnostic tests using RDTs. Since 2005, and especially following WHO’s 2006 Treatment Guidelines, Global Fund financing has enabled substantial increases in RDT distribution and use. The recent increase in planned and actual RDT procurements in 2009 and 2010 suggests that RDT usage probably continued to increasing sharply over 2011 and next years.

Adoption of a complex health innovation involves series of changes in behavior and practice [20]. Making resources available to procure RDTs is only the first step. An efficient delivery system is vital to distribute diagnostic kits to users and ensure their timely use within the shelf life. Effective RDT usage is also influenced by the readiness of health facilities, staff trained in RDT use and regular supply of RDTs, especially during the high transmission seasons, together with ACTs and other essential drugs to treat non-malaria febrile cases. Health personnel should be well trained to perform RDTs, interpret results, and prescribe treatment accordingly. Besides sustained training and capacity building, appropriate incentives to use RDTs may be needed, as many health staff in malaria endemic countries are still using presumptive treatment when managing such cases, or questioning RDTs specificity and/or sensitivity as a reason for continuing with old practices [11]–[13], [21]. Clinicians have to learn to manage negative results and to evaluate cases carefully to consider other possible causes of malaria-like symptoms, in addition to prescribing anti-malarial drugs only when warranted.

Although impressive, the observed increase in number of RDTs procured still falls short of estimated need. The Roll Back Malaria Partnership [16] estimated a global annual need of 1.6 billion suspected malaria cases requiring diagnosis in 2010 alone – assuming that all fever cases in all endemic countries would seek care. To cover 50% of these fever cases, 800 million RDTs would have been needed in 2010 alone to confirm or exclude malaria, well above the 24.5 million RDTs procured or 48.97 million RDT kits planned procurements in Global Fund-supported programs in 2010. The other major external funder of malaria programs globally, PMI, had procured 25.1 million RDTs cumulatively by 2010 [22], bringing the total externally financed RDT purchases to just below 50 million in 2010– much lower than the estimated global need.

We found low ratios of RDT procurements to ACT procurements in programs supported by both the Global Fund and the PMI. In 2010, Global Fund recipients procured one RDT per 2.94 ACTs (24.5 million RDTs versus 72.1 million ACTs), and PMI one RDT per 3.07 ACTs (13.3 million RDTs versus 41.0 million ACTs). Although still low, these ratios have increased from 2009, when they were one RDT per 3.9 ACTs for Global Fund, and one RDT per 3.5 ACTs for PMI. In line with relatively low RDT procurement, only 5% of the budget of Global Fund malaria grants was allocated to malaria diagnosis in 2010. Similarly, in malaria programs supported by the PMI, diagnosis covered only 7.5% of budgets during fiscal year 2011 [23].

These results indicate that there is room to improve budget allocation in Global Fund –supported malaria programs, optimizing the effectiveness and value for money in case management, by more balanced allocations and priority scale-up for parasitological diagnosis, especially in the highest-endemic African countries. Between 2002 and 2010, the Global Fund financed proposals based on country demand. This has generally resulted in an equitable distribution of grant disbursements by disease burden, but not necessarily optimized allocations among interventions within each disease program. The Global Fund’s 2012–2016 Strategy ‘Investing for impact’, recent revisions in country eligibility and prioritization criteria, and refined requirements for counterpart financing to disease programs by all supported governments now facilitate better targeting of Global Fund funding to essential highest-impact interventions and to countries with the highest continuing need [24]. In June 2012, the Global Fund became signatory to WHO’s ‘Test Treat Track initiative [25]. As a consequence, all malaria grants that include case management activities must now include a pragmatic and realistic approach to diagnostic scale-up to be approved.

The analysis is not without limitations. One limitation is the completeness of grant-reported data. Regarding program implementation, indicators included in grant performance frameworks reflect only selected activities with large budgets that contribute critically to the objectives and goals of the proposal. RDT services delivered are not always fully reported by all programs receiving RDT support, so that the programmatic results presented must be considered minimum estimates.

Similarly, PQR reporting of RDT procurements is probably not complete across all grants and programs, as reporting is enforced only at the time that a grant completes its Periodic Review after 2 years of grant implementation. Our exclusion of some price outliers from the PQR-based analysis contributes further to the procurement figures representing a lower-bound estimate. For microscopy-based diagnosis, indicator data on microscopes procured, health facilities equipped with microscopes and health workers trained to use microscopes are at present not systematically aggregated in the Global Fund’s portfolio-level data system, because of known limitations in completeness and comparability among grants. Reporting on the indicator ‘*RDTs and microscopy slides read’* certainly does not cover the majority of microscopy-based diagnoses delivered in programs whose grants did not support RDT roll-out. For these reasons, the data presented cannot be used to quantify the flow from RDT procurements to distribution and usage, or the balance between tests used and personnel trained.

Moreover, the analysis presents numerical results on the extent of Global Fund support to the adoption of 2006 and 2010 WHO Malaria Treatment Guidelines, but it does not systematically compare these results with the corresponding need to realize universal diagnostic access, such as population at risk, demand in terms of suspected malaria or fever cases, health staff requiring training or health facilities to be equipped.

Despite these limitations, the present analysis is the first, most comprehensive systematic triangulation of Global Fund support to rolling-out RDT-based diagnosis through all stages in the chain of an innovative policy implementation. As the Global Fund transitions to a new model of financing health programs based on ‘informed demand’ that prioritizes proven highest-impact interventions, and strategic programming or re-programming to replace the more ‘passive disbursement’ that characterized the period 2000–2009 [24], investments in RDTs should increase. Increased RDT investments are critical to realize the RBM and WHO target of universal coverage of parasitological diagnosis before treatment initiation and to achieve the global goal of reducing malaria deaths to near-zero by 2015 [26].

## Supporting Information

Box S1 **The Global Fund business model and programmatic results.**
(DOC)
