# Supplementary material for: Adoption of Rapid Diagnostic Tests for the Diagnosis of Malaria, a Preliminary Analysis of the Global Fund Program Data, 2005 to 2010
Source: PLoS One. 2012 Aug 27;7(8):e43549. doi: 10.1371/journal.pone.0043549 (PMC3428362; doi:10.1371/journal.pone.0043549)
Supplement: Box S1 — The Global Fund business model and programmatic results. (DOC) [file pone.0043549.s001.doc]

**Box S1. The Global Fund business model and programmatic results**

The Global Fund’s business model for grant making and management includes performance based funding as one of its core principles. For each grant, the Global Fund and the Principal Recipient (PR) sign a grant agreement serving as the legal contract, which includes a Performance Framework, proposed by the PR and negotiated between the Global Fund and the PR, following the program logic framework of the proposal. A Performance Framework typically contains 2 to 5 impact and outcome indicators measuring the progress towards the goal (s) and objectives defined in the grant proposal, and aligned with national and/or international disease control targets. The Performance Framework also contains up to 15 programmatic indicators that measure progress towards implementation targets. At each disbursement request, results on programmatic indicators are reported and assess against their respective targets. A rating is given that quantifies performance on each indicator, resulting in an overall rating calculated across all indicators. Disbursement amounts are decided based on the performance over the reporting period, which is usually 6 months.

In addition to the results reported against the targets of indicators included in the Performance Frameworks, the Global Fund also has the following program data,

- Finance, including budget, disbursement and expenditure, with breakdown by service delivery areas
- Procurement, including procurement plan, price and quality reporting, with breakdown by major categories of health products.
